# Dr. Simpson’s Morphia Suppositories

**Published:** 1857-05

**Authors:** 


*Dr. Simpson's Morphia Suppositories.*—Mr. Spencer Wells has introduced into use at the Samaritan Hospital, a form of morphia suppository, used with great advantage by Dr. Simpson of Edinburg. Mr. Wells has found it a most convenient form of suppository after operations on the vagina, rectum, uterus, or perineum of women, both in hospital and private practice, and especially so after operations on the male genito-urinary organs, as lithotrity, in cases of retention of urine, irritable structure, &c., and after division of fistula in ano, or the removal of piles or prolapsed mucous membrane of the rectum by the ecraseur. They act much more efficiently than the soap and opium in common pill use as a suppository, and are seldom or never expelled from the rectum after their introduction above the sphincter. They are made extremely well by Messrs. Duncan and Flockhart, of Edinburgh, and supplied by them at a very reasonable rate, of various strengths. But as they are likely to come into more general use, we append the formula on which they are prepared. The following is for the ½ grain suppository: Take the acetate of morphia, 6 grains; sugar of milk, 1 drachm; simple cerate, half a drachm, or as much as may be sufficient to make a proper consistence, and divide the mass into twelve suppositories. Then dip each suppository into the following mixture, to form a coating: Take of white wax 1 part, lead plaster 2 parts; melt together. The best way is to insert a needle into the apex of the suppository, dip it into the melted wax and lard, and immediately afterwards into cold water to harden it before it loses it shape. The shape is conical, like a pastille. It is easily introduced by the finger, or more neatly by the ordinary ivory suppository syringe. Mr. Coulson has also used these suppositories lately in several lithotrity cases, and has found them of the greatest benefit in allaying the irritation which often attends the passage of the fragments of calculi through the urethra.—*Med. Times and Gaz.*, Feb. 7, 1857.

